# Supplementary material for: Comparative multiomics analysis of cell physiological state after culture in a basket bioreactor
Source: Sci Rep. 2022 Nov 23;12:20161. doi: 10.1038/s41598-022-24687-4 (PMC9686226; doi:10.1038/s41598-022-24687-4)

W_023666075.1:4095608:4099567:-@NW_023666075.1:4095608:4099570:-@NW_023666075.1:4104024:410407[illegible]

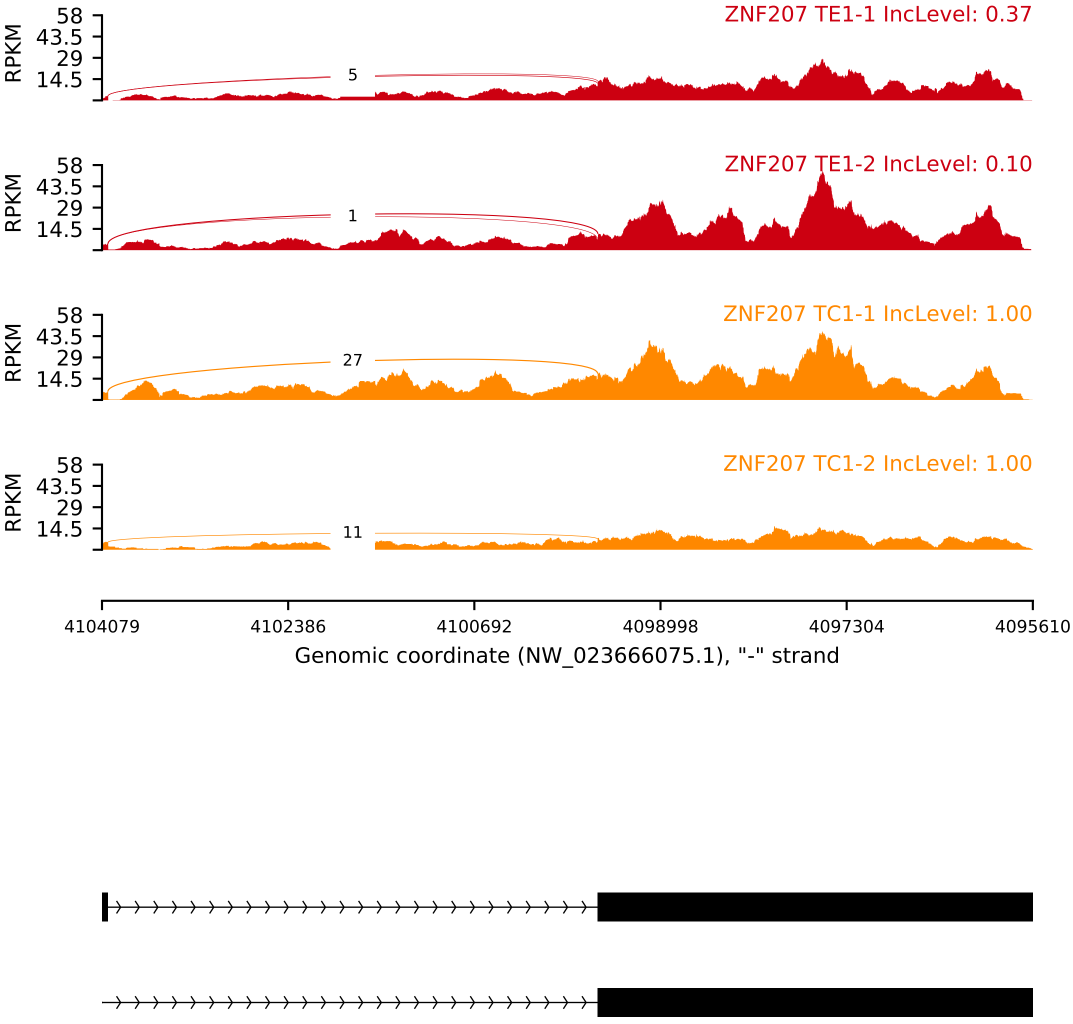

Supplement: Supplementary file 1 — Supplementary Information 1. [file 41598_2022_24687_MOESM1_ESM.zip › raw data/Transcriptomic raw data/7.AS/2.dasplot/TE1vsTC1/A3SS/1_ZNF207_NW_023666075.1_4095608_4099567_-@NW_023666075.1_4095608_4099570_-@NW_023666075.1_4104024_4104078_-.pdf]
